# Supplementary figures and images for: Conformational plasticity and dynamic interactions of the N-terminal domain of the chemokine receptor CXCR1
Source: PLoS Comput Biol. 2021 May 20;17(5):e1008593. doi: 10.1371/journal.pcbi.1008593 (PMC8172051; doi:10.1371/journal.pcbi.1008593)

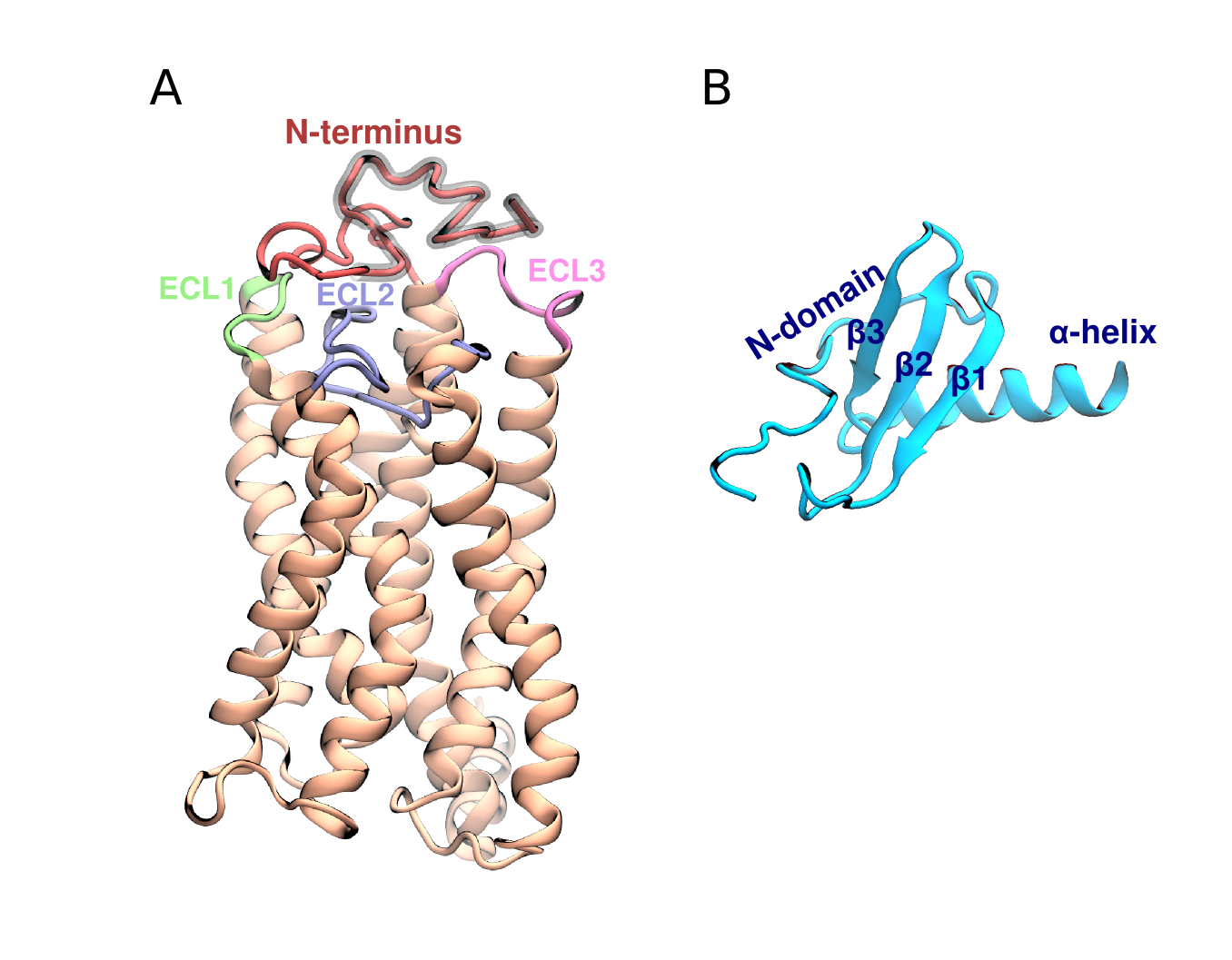

Supplement: S1 Fig — NMR structure of (A) CXCR1 (PDB ID: 2LNL) with unresolved region of the N-terminus highlighted as a gray tube and (B) interleukin-8 (PDB ID: 1IL8). Extracellular domains of CXCR1 viz. ECL1, ECL2, ECL3 and N-terminus are colored as green, light blue, pink and red, respectively. IL8 is shown in cyan, with each region labeled. (TIF) [file pcbi.1008593.s001.tif]

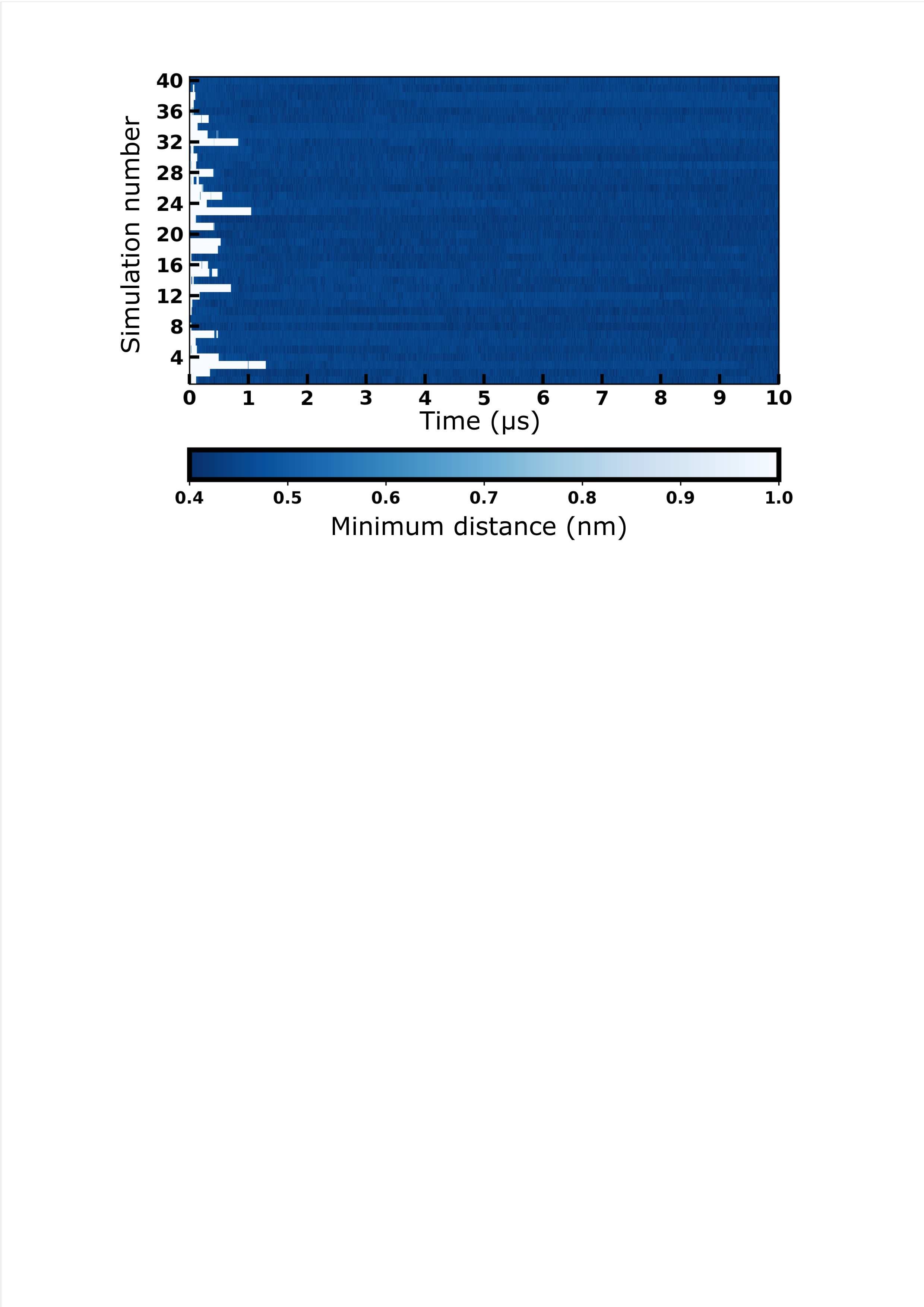

Supplement: S3 Fig — Minimum distances between IL8 and CXCR1 are plotted as a function of time for forty simulations. The white and blue stretches represent unbound and ligand-bound regimes, respectively. (TIF) [file pcbi.1008593.s003.tif]

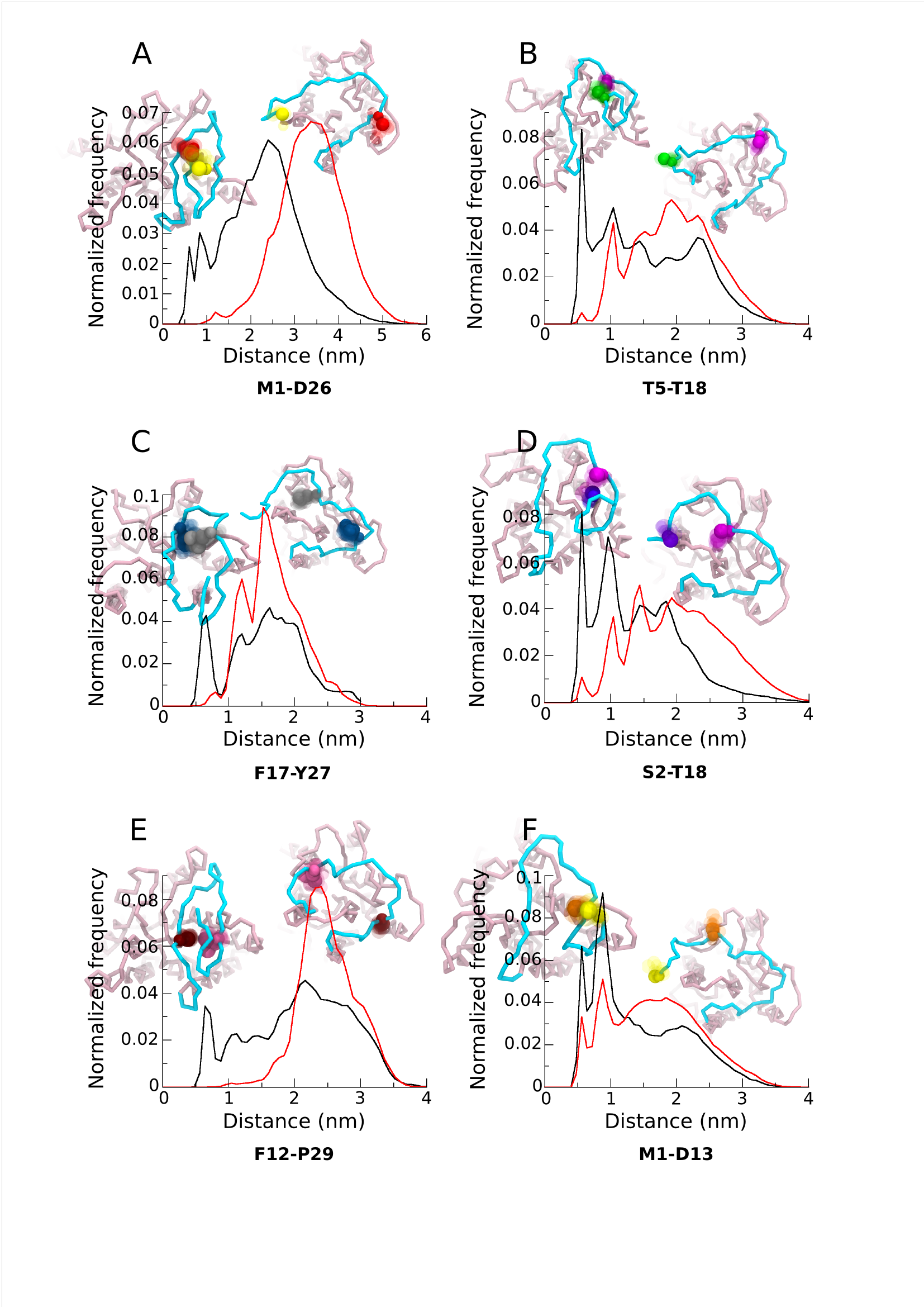

Supplement: S4 Fig — Normalized population histograms of distances between center of masses of side chains of residue pairs (A) Met1(yellow)-Asp26(red), (B) Thr5(green)-Thr18(magenta), (C) Phe17(gray)-Tyr27(blue), (D) Ser2(violet)-Thr18(magenta), (E) Phe12(pink)-Pro29(maroon) and (F) Met1(yellow)-Asp13(orange). The black lines represent apo-CXCR1 and red lines represent CXCR1-IL8 simulations. Representative top-view snapshots from apo-CXCR1 (left) and IL8-bound (right) CXCR1 N-termini are displayed on top of each histogram. The N-terminal region is shown in cyan and the rest of the CXCR1 receptor is in pink. (TIF) [file pcbi.1008593.s004.tif]

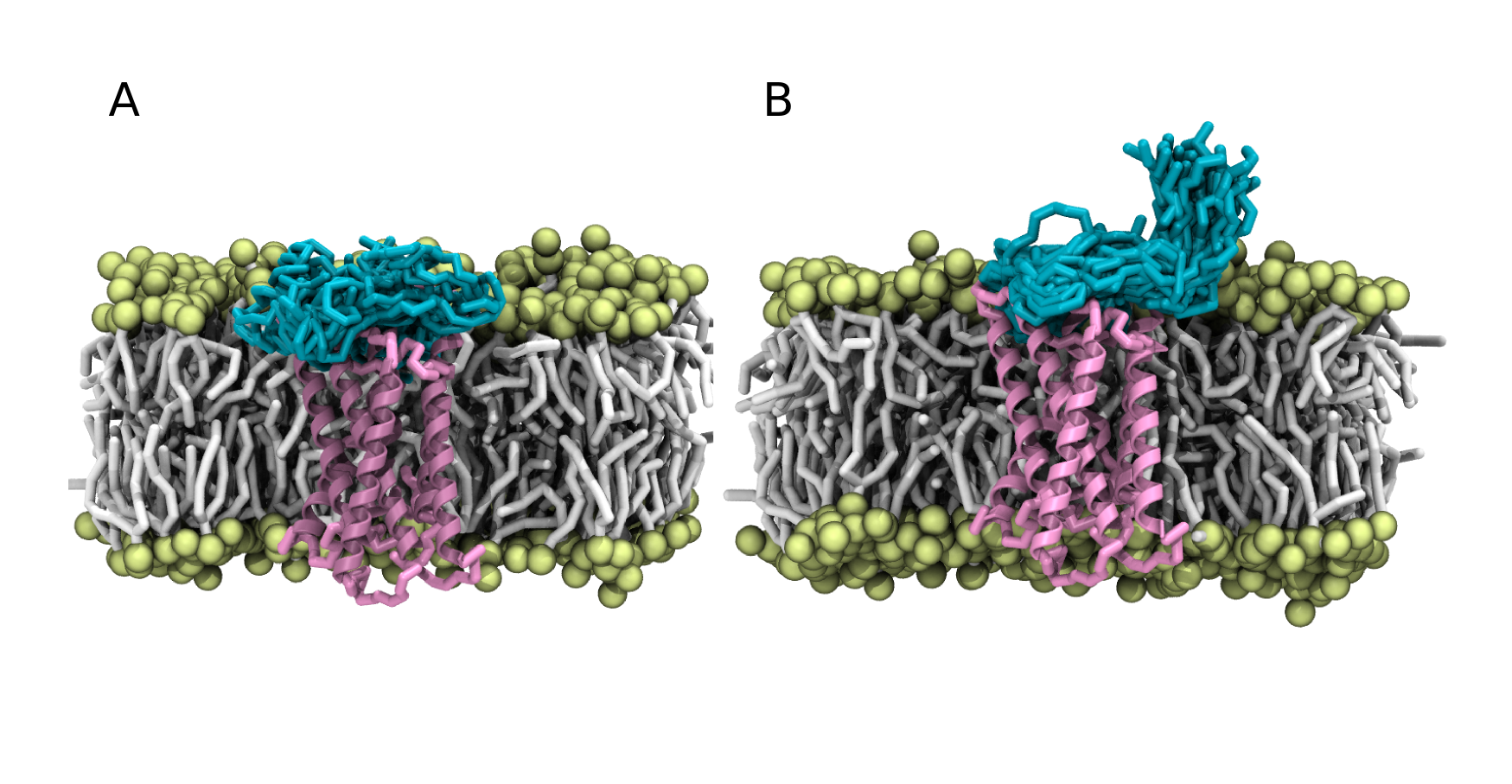

Supplement: S5 Fig — The most populated conformations (side view) are shown for the apo- (left) and ligand-bound (right) forms of the receptor. The N-terminal region is shown in cyan and the rest of the receptor is in pink. The phospholipid headgroups are represented as yellow beads and acyl chains are in gray. (TIF) [file pcbi.1008593.s005.tif]

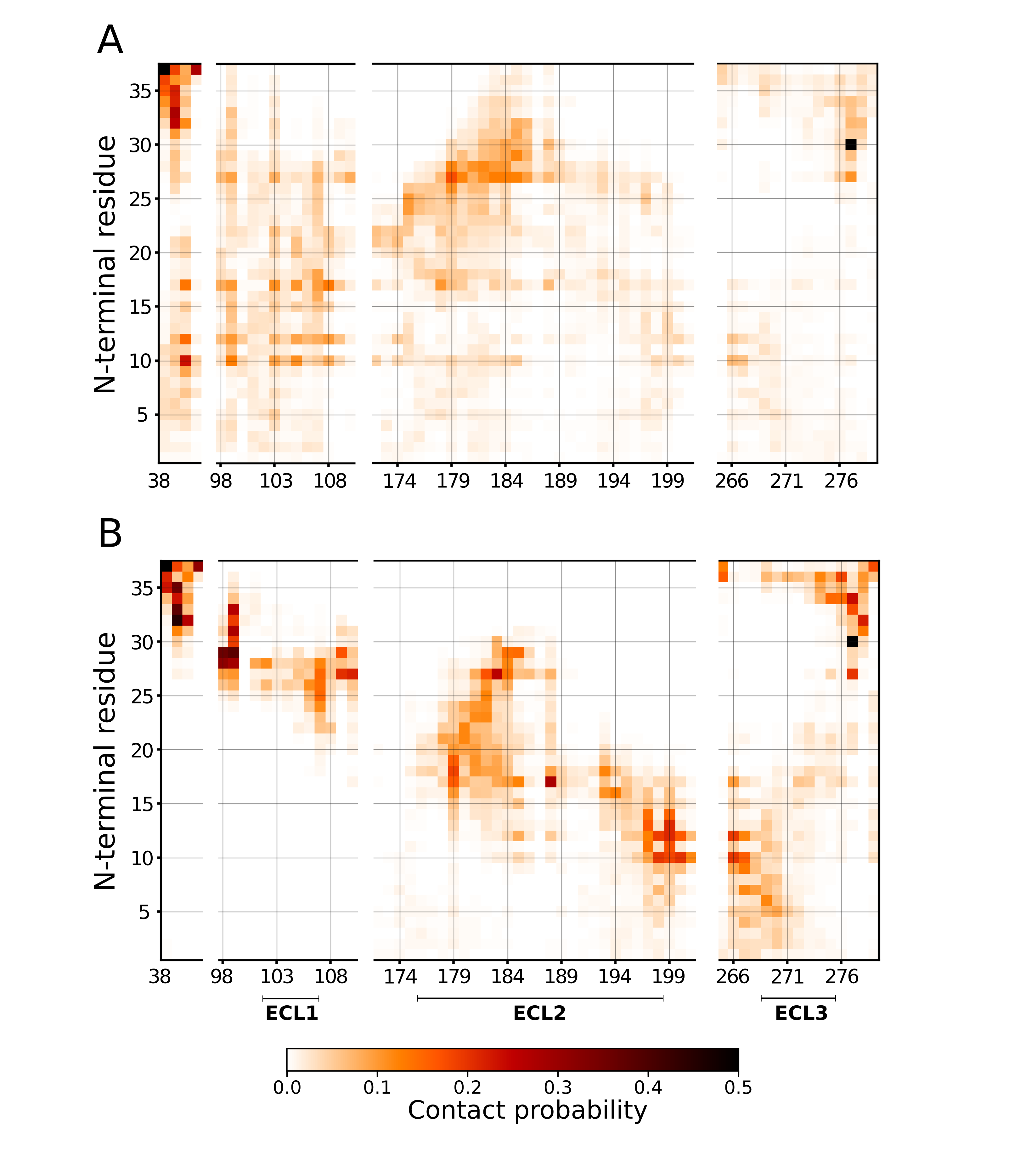

Supplement: S6 Fig — Contact maps of the N-terminal region in the C-shaped conformation interacting with the extracellular domains of the receptor for (A) apo-CXCR1 and (B) IL8-bound CXCR1. (TIF) [file pcbi.1008593.s006.tif]

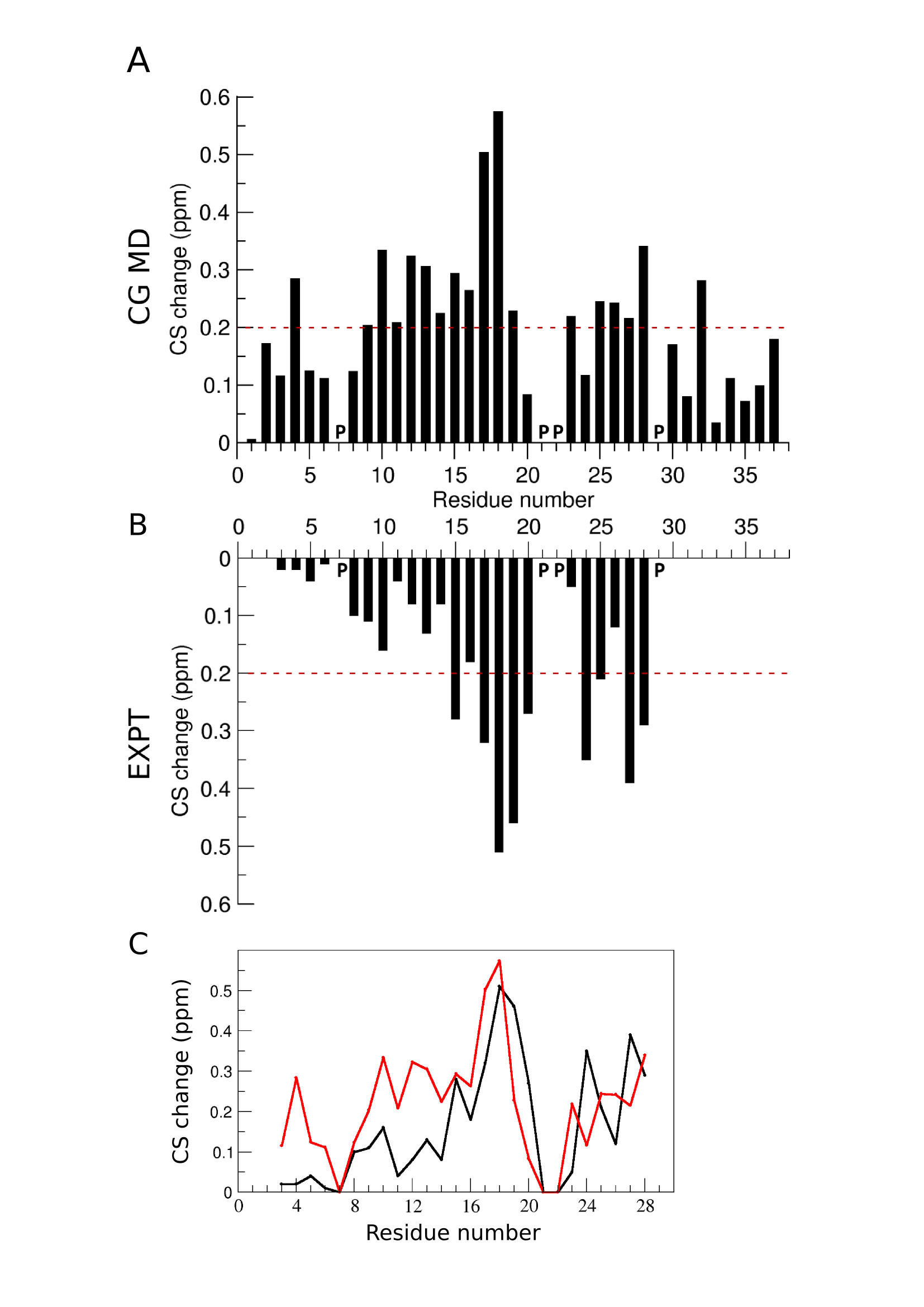

Supplement: S7 Fig — (A) Predicted and (B) experimental chemical shift changes in the N-terminal region between the apo- and ligand-bound states. (C) Chemical shift differences plotted as a line graph for experimental (black) and predicted (red) values. (TIF) [file pcbi.1008593.s007.tif]

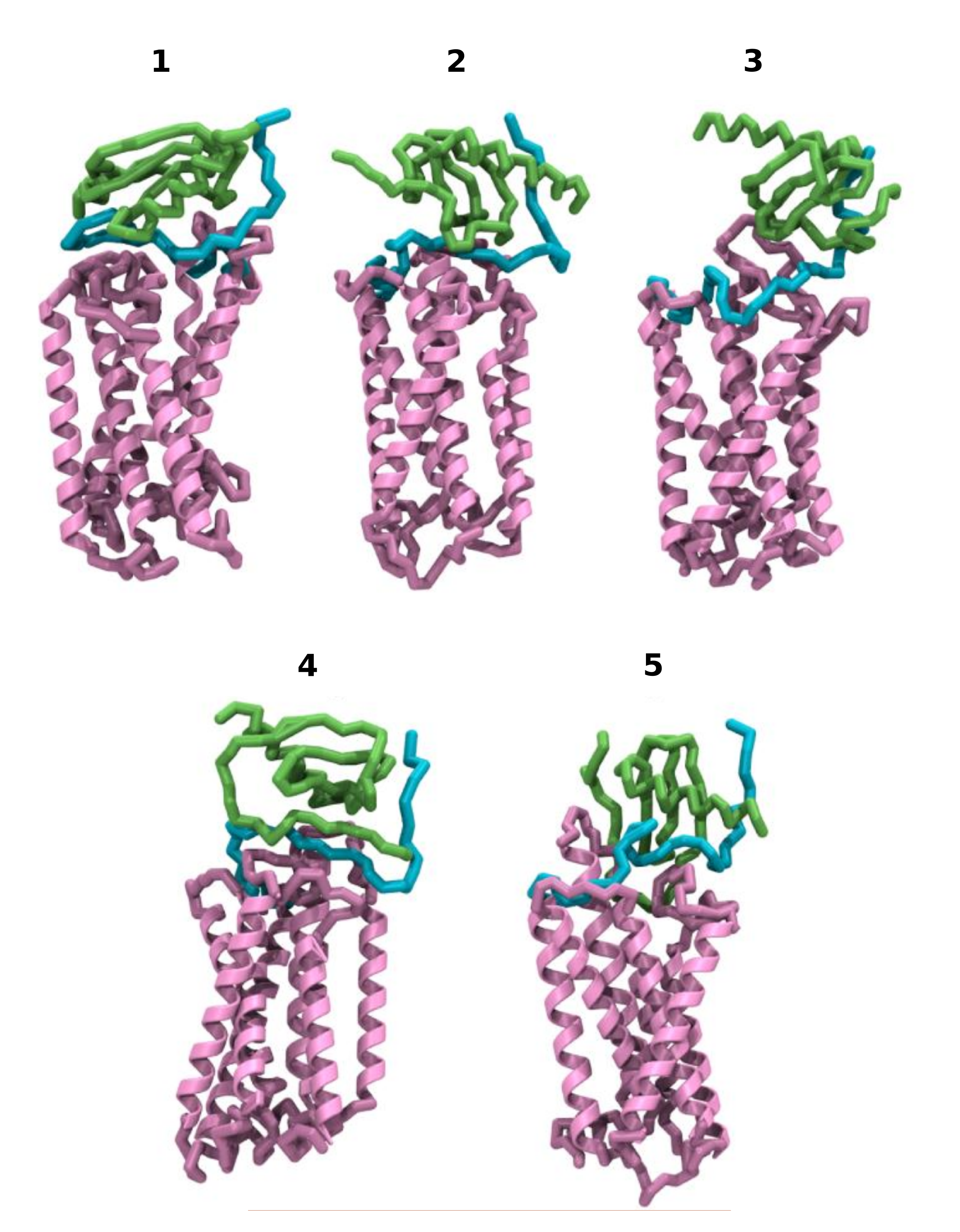

Supplement: S8 Fig — The five dominant binding modes (1–5) are shown. The receptor is shown in pink, the N-terminal region is shown in blue, and the IL8 is represented in green. (TIF) [file pcbi.1008593.s008.tif]
