# Supplementary material for: Conformational plasticity and dynamic interactions of the N-terminal domain of the chemokine receptor CXCR1
Source: PLoS Comput Biol. 2021 May 20;17(5):e1008593. doi: 10.1371/journal.pcbi.1008593 (PMC8172051; doi:10.1371/journal.pcbi.1008593)
Supplement: S2 Fig — (A) Residue-wise contact probabilities of the N-terminal region are plotted for apo-CXCR1 in coarse-grain simulations (upper diagonal) and atomistic simulations (lower diagonal), averaged over all simulation sets. The color bar displays probability of interactions between each residue-pair. (B) A plot of secondary structures of the N-terminal residues along the atomistic simulation trajectory. The secondary structure was calculated according to the DSSP algorithm [1]. White, red, yellow, black, green, blue and gray stretches represent coil, β-sheet, turn, β-bridge, bend, α-helix and 310-helix, respectively. (PDF) [file pcbi.1008593.s002.pdf]

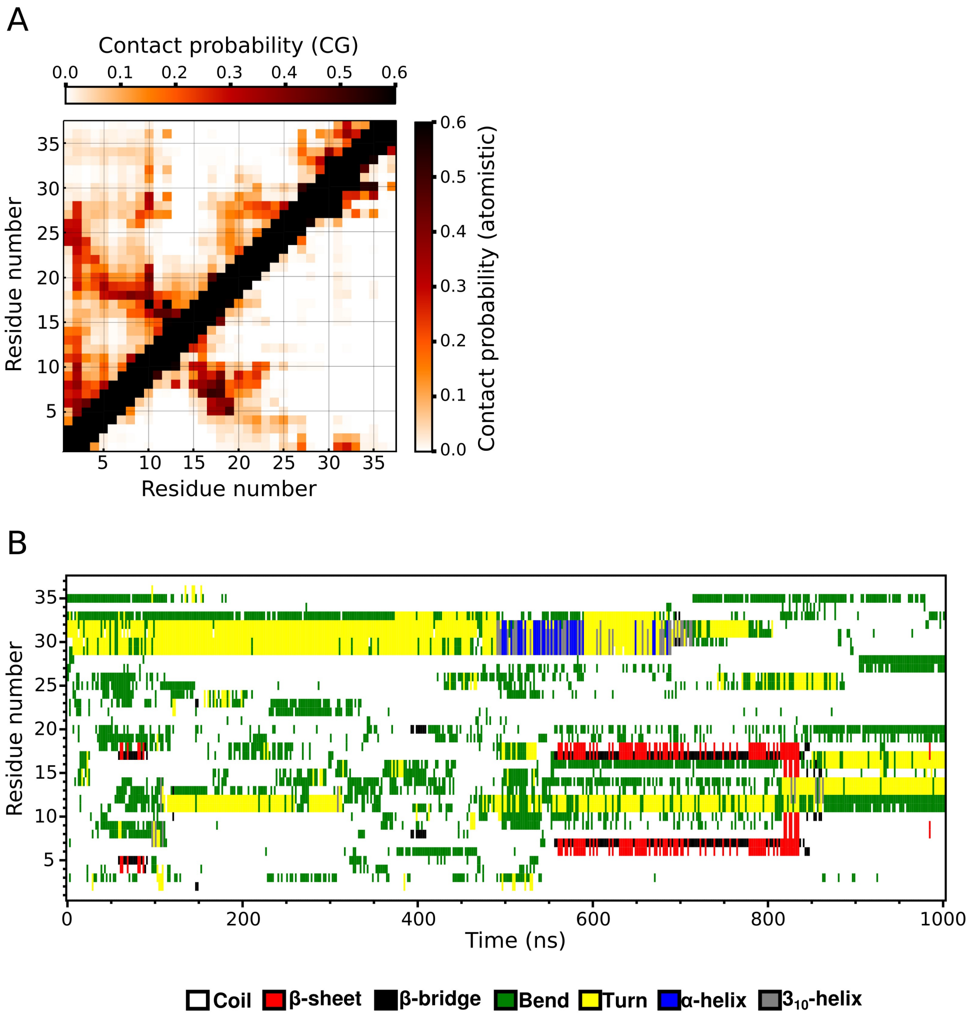

References

1. Kabsch W, Sander C. Dictionary of protein secondary structure: pattern recognition of hydrogen-bonded and geometrical features. Biopolymers 1983;22:2577-2637.
