# Supplementary material for: Conformational plasticity and dynamic interactions of the N-terminal domain of the chemokine receptor CXCR1
Source: PLoS Comput Biol. 2021 May 20;17(5):e1008593. doi: 10.1371/journal.pcbi.1008593 (PMC8172051; doi:10.1371/journal.pcbi.1008593)
Supplement: Attachment — Submitted filename: Chattopadhyay response to reviewers.docx [file pcbi.1008593.s011.docx]

April 12, 2021

Dr. Turkan Haliloglu
Associate Editor
PLOS Computational Biology

Dr. Nir Ben-Tal
Deputy Editor
PLOS Computational Biology

Dear Drs. Haliloglu and Ben-Tal:

Thank you for your letter of 26^th^ January and the accompanying reviewers’ comments on our manuscript titled “***Conformational plasticity and dynamic interactions of the N-terminal domain of the chemokine receptor CXCR1***” by S. Kharche, M. Joshi, D. Sengupta and myself (**PCOMPBIOL-D-20-02189**). We appreciate the reviewers’ comments and suggestions, and have taken them into account in appropriately revising the manuscript.

Our response to the reviewers’ comments and the specific changes made are summarized below and are highlighted in the revised manuscript (*reviewers’ comments on top in italics*):

**Queries from Editors**

1.	*The authors may want to correlate their simulations with evolutionary data (e.g., using ConSurf), where the premise is that biologically important sites that are mechanistically critical would often be highly conserved. It may further consolidate the simulations. But please feel free to ignore.*

**Our response:** The N-terminal domain of chemokine receptors are quite diverse, even within a subfamily. We tested the ConSurf website; however, the algorithm requires longer sequence stretches and we were unable to analyze just the N-terminal domain by itself. The sequence of CXCR1 corresponding to the PDB structure (2LNL) was plugged into the ConSurf database to search homologs against the sequence databases UniProt and UniRef90 (Fig R1, see below). After applying a threshold cutoff, 300 unique homologs were selected for multiple sequence alignment and conservation score calculations. However, we could not retrieve the conservation profiles for the first 19 residues of the N-terminus, as the ConSurf database could not process that sequence.

**Fig R1.** Conserved sequence scales in the CXCR1 receptor. The residue numbering starts from residue 21.

In the future, we plan to analyze co-evolution of the receptor/ligand pairs and reanalyze the N-terminal sequence conservation from the suggested perspective. As mentioned above, it is difficult to reconcile the simulation data with sequence data at this stage.

**Reviewer #1**

1*.	The article “Conformational plasticity and dynamic interactions of the N-terminal domain of the chemokine receptor CXCR1” by Shalmali Kharche, Manali Joshi, Amitabha Chattopadhyay, Durba Sengupta presents a study of the interaction between the CXC chemokine receptor 1 and interleukin-8 by molecular dynamics. This is an important subject, well introduced, and the results are generally very convincing. The main results are: 1) the dynamics of the N-terminal region undergo a conformational selection upon ligand binding, i.e. it switches from being unstructured in the apo-receptor to a C-shaped conformation upon ligand binding. 2) The comparison with NMR data shows that the results are generally supported by experiment. Interestingly the authors noticed that the chemical shifts are at least partially due to the change in conformation, calling for caution in the interpretation of chemical shift experiments. This second result is interesting per se.*

**Our response:** We thank the reviewer for the positive comments on our manuscript.

**Major points**

2.	*One limitation is that the authors use clustering but neither detail the underlying methods nor present the clustering itself. It would be useful for the reader to see the clustering, even as supplementary material. One potential question is: How many clusters should be considered?*

**Our response:** The majority of the data presented in the manuscript depicts either a complete sampling (*e.g.*, Fig 2b) or an average representation (Fig 2c). The clustering was done for (i) identifying a reference conformer for projecting the entire phase space sampled (Fig 3) and (ii) to reduce the phase space for the backmapping protocol (Fig 5). In Fig 3, the entire phase space was calculated with respect to residues 1-26 and RMSD to this reference structure. The populations projected on the 2D surface were binned (0.1 nm) and averaged. The three clusters that were observed (I, II, III) are a direct outcome of the projection (without prior clustering) and correspond to the maxima of the population histograms of the conformers sampled over the entire set of trajectories, thereby directly reflecting the corresponding minima in the underlying energy landscape. The clustering was performed using the GROMACS utility gmx cluster using the original GROMOS algorithm implemented in it (Daura *et al.* 1999) using the N-terminal region to fit the conformers. In the second step, we used these clusters to identify the main conformers (total 20 *apo* + 40 ligand-bound) to map to the atomistic representation and for estimating the chemical shifts. In the current approach, each backmapped structure needs to be minimized and equilibrated individually and to backup the entire trajectory is rather difficult. As a result, we have backmapped only these 60 structures and minimized and equilibrated these 60 structures through short molecular dynamics simulations. We have now updated the Methods section (p.20-21 in the revised manuscript) to include the details of the clustering algorithm.

3.	*In principle, the authors could even consider PCA in terms of analysis of their trajectories, given that they appear to be converged.*

**Our response:** Since we wanted to capture the main differences between the conformational states in the *apo*- and ligand-bound receptor, we attempted to project the populations sampled on the vectors with the maximal difference. As suggested by the reviewer, we have now performed a PCA analysis and projected the sampling in the simulations of the *apo*-receptor and the receptor with the ligand bound (Fig R2, see below). Overall, we see an increased sampling of the phase space in the *apo-*receptor, compared to the ligand-bound receptor, but it is much less clear compared to the 2D projection in the manuscript (Fig 4 in the revised manuscript). It appears that the first two principal component vectors are unable to capture the differential dynamics, in part since the dynamics in the *apo*-receptor is not easily captured by the first two component vectors and a larger phase space could be needed to better analyze these differences.

**Fig R2.** 2D projection of principal component vectors 1 and 2 for (a) *apo-* and (b) IL8-bound receptor simulations.

4.	*It would be useful to see the different binding modes (Fig 6). It would also be good to know how many times each one occurred. Does it depend on the starting orientation? Incidentally it is unclear whether, when binding occurred more than once, whether rebinding occurred with the same binding mode. Finally, one may wonder whether the data presented in figure 2c depends on the binding mode.*

**Our response:** To analyze the different binding modes, we have plotted the binding modes over time (Fig R3, see below). We observe a very fast (nanosecond timescale) interconversion between the different binding modes, especially nodes 1-2, 1-3 and 4-5. The nanosecond timescale interconversion suggests that there is no direct correlation between the initial binding event and the final modes, although some transitions are more frequent than others. We believe that much longer sampling and a Markov state model based analysis will be needed to rigorously identify and estimate stationary populations of key macrostates, predicting eventual binding constants, (on and off) rate constants and the pathways (as also suggested by Reviewer #2). However, this is out of scope of the current study and will be considered in the future.

**Fig R3.** Time course of binding of IL8 to CXCR1 during the course of the simulations. The binding modes are color coded 1-5, with white (mode 6) corresponding to any non-specific binding event. The panel on the left corresponds to the first initial binding mode (t*=0). We observe a very fast (nanosecond timescale) interconversion between the different binding modes, especially nodes 1-2, 1-3 and 4-5.

5.	*It is not completely clear how the contacts presented in Fig 3 were selected. Why not mention residue 10 and its interactions with residues 17, 20 and 29? or the interaction between 12 and 17? The interaction between 12 and 29 is also ignored whereas it is presented in Fig S3. This is important because the interaction between residues 1 and 26 is then used as a collective variable and one may wonder whether the picture given by fig 4 could be different if one other residue pair would have been considered.*

**Our response:** The distances were chosen from the contact maps with the maximal difference between the *apo*- and ligand-bound receptor conformations (Fig 3 in the revised manuscript). In general, the close contacts are higher in the *apo*-receptor and reduced in the ligand-bound state, and indeed other pairs could have been considered. If we consider residues 10-17, 10-20, 10-29 (Fig R4, see below), the number of close contacts for the 10-17 pair are reduced but not completely absent, unlike the 10-20 and 10-29 pairs. These two perhaps could also be used as a reporter. However, using residues Met1-Asp26 also reports on the overall opening/closing of the N-terminal. The interactions of Met1 reduces beyond Asp26, and the dynamics of that stretch is constrained due to spatial proximity to secondary structure of helix I. In fact, we projected the population space on the radius of gyration (Rg) of the N-terminal region (residues 1-26), instead of the Met1-Asp26 distance (Fig R5, see below) and observed the same trend in both. The parameter Met1-Asp26, therefore, reports not just on that particular residue pair but on the larger conformational changes associated with opening/closing of the N-terminal domain.

**Fig R4.** Normalized population histograms of distances between center of masses of side chains of key residue pairs. The black lines represent *apo*-CXCR1 and red lines represent CXCR1-IL8 simulations.

**Fig R5.** Population density map of the conformations sampled by the N-terminal region plotted as a function of backbone RMSD of the N-terminal region and (top) distance between residues (Met1 and Asp26) and (bottom) radius of gyration (residues 1-26) for (a,c) *apo*-CXCR1 and (b,d) IL8-bound CXCR1. The top panel is taken from Fig 4 in the revised manuscript.

6.	*The comparison with experimental data is an important aspect of the study and should be made as quantitative as possible. If the authors cannot get the numbers from the authors of the NMR experiments perhaps they could make the comparison more stringent by playing on the threshold, in order to reduce the number of residues with high computed chemical shift.*

**Our response:** We have plotted the experimental values from the NMR data as a bar plot and as a line plot (Fig R6, see below). Overall, the trends match well, as observed in the qualitative analysis of important residues depicted earlier. However, the quantitative differences are high - as perhaps could be expected for calculations from a coarse-grain simulation being backmapped to an atomistic one. Overall, the trend is similar in the experimental and simulation values. We have included this figure as S7 Fig in the revised Supporting Information.

**Fig R6.** Chemical shift perturbations induced in the N-terminal region interacting with IL8. (a) Predicted and (b) experimental chemical shift changes in the N-terminal region between the *apo*- and ligand-bound states. (c) Chemical shift differences plotted as a line graph for experimental (black) and predicted (red) values.

**Minor points**

6.	*For the conformational landscape presented in figure 4 it would be interesting to have more information about the clustering. It would also be interesting to have information about the dynamics, i.e. do the authors observe fluctuations between the basins during the simulations. As for the structures shown, it would be interesting to have also a view in the membrane plane.*

**Our response:** To better characterize the structures, we have added a view in the membrane plane (Fig R7, see below). In addition, we have added a side view of the receptor as S5 Fig in the revised Supporting Information.

To analyze the interconversion between the different states, we generated a scatter plot of a few simulation sets (Fig R8, see below). Both interconversion between states and sampling of a single state by the receptor were observed. The simulation set represented in black predominantly samples state 1. However, the simulation set represented in green appears to interconvert between states 1 and 3.

**Fig R7.** Conformational landscape of the N-terminal region of CXCR1. Population density map of the conformations sampled by the N-terminal region plotted as a function of backbone RMSD of the N-terminal region and the distance between side chains of two representative residues (Met1 and Asp26) for (a) apo-CXCR1 and (b) IL8-bound CXCR1. The most populated conformations (side view) are shown for the *apo*- (left) and ligand-bound (right) forms of the receptor. The N-terminal region is shown in cyan and the rest of the receptor is in pink. The phospholipid headgroups are represented as yellow beads and acyl chains are in gray.

**Fig R8.** Conformational landscape of the N-terminal region of CXCR1. (a) Population density map of the conformations sampled by the N-terminal region plotted as a function of backbone RMSD of the N-terminal region and the distance between side chains of two representative residues (Met1 and Asp26) for *apo*-CXCR1. (b) Scatter plots of a few representative simulation sets (colored by set #).

7.	*The receptor is known, as mentioned by the authors, to form dimers. It would be interesting for the reader to have an idea of whether the results could be altered in the dimer. For instance, the authors could discuss/comment on the potential overlap between the dimerization interface and the interface with IL8 or area explored by the Nter.*

**Our response:** We thank the reviewer for the interesting comment - indeed the dimerization of the receptor could alter ligand binding. However, the 1:1 stoichiometry (1 receptor:1 ligand) has been reported to probably be the predominant signaling form. The dimer interface of CXCR1 is not yet known, but the crystal structure of CXCR4 suggests a dimer interface formed by transmembrane helices IV and V. Since transmembrane helix I faces outward, the dynamics of the N-terminal domain would not be directly constrained. We have extended the discussion in the revised manuscript accordingly, as follows: “*An important point to consider would be the dimerization of the receptor and the ligand could alter ligand binding. However, the 1:1 stoichiometry (1 receptor:1 ligand) has been reported to probably be the predominant signaling form^24-26^.*” (lines 455-458, p.18 in the revised manuscript).

8.	*Given that interaction with the membrane is known to affect the function of the receptor, and the nter to interact with the membrane, it would be worth commenting/discussing on the potential impact of having the membrane represented by a pure POPC bilayer.*

**Our response:** We thank the reviewer for the comment. Indeed, that is the next step and we are currently working on a more complex membrane. In addition, we have touched upon this important aspect in the revised manuscript, as follows: “*Another limitation of the current work is the simple membrane model considered. However, several of the experimental studies (fluorescence, NMR) have been performed in model membranes or even detergent micelles. The main salient features of the interaction are not suggested to differ, although the binding would be modulated overall.*” (lines 458-461, p.18 in the revised manuscript).

**Reviewer #2**

1.	*The conformational dynamics of N-termindal domain of CXCR1 with interleukin-8 (IL-8) has been explored using state-of-the-art computer simulation approaches by Kharche and coworkers. The authors first explored the dynamics of apoprotein using coarse-grained simulation and then validated its prediction against all-atom simulation. Subsequently, the model investigated the protein-protein association dynamics using coarse-grained simulation for extensive period of time and pin-pointed the key locations of the association process. The manuscript is well-written and most of the analysis is clear. The reviewer has enjoyed reading the draft and found it commendable to explore recognition process via coarse-grained simulation, an emerging approach. The referee believe that this manuscript is well-suited for publication in PLOS com. biol.*

**Our response:** We thank the reviewer for appreciating the relevance and implications of our work.

2.	*‘The final bound complex’ is a vague term in this work. In typical protein-ligand binding simulation, generally the ‘binding simulation’ are terminated after the bound pose agrees with the crystallographic pose. Here, In absence of experimentally known pose, the criteria for termination of this long simulation should be properly justified.*

**Our response:** We thank the reviewer for pointing out that the term is ambiguous in the current context. We have used the term to refer to the bound complex sampled at the end of the simulation sets. Indeed, in the absence of a crystal (or cryo-EM) structure and based on our own work that suggests a high degree of dynamics, we have now replaced the term with “CRS1 bound complex” (line 193, p.8 and lines 212-213, p.9 in the revised manuscript). Since we have focused on CRS1, the first site of binding, we have terminated the simulations after a microsecond timescale sampling of this site. Subsequently, the CRS2 site is sampled, which includes further contacts from the receptor lumen and is suggested to occur at much longer timescales.

3.	*The author’s claim that the the dynamic complex sampled by the simulation is validated by NMR data is a bit of stretch. The question is, in absence of a crystallographic or cryo-EM data, the reviewer is not very convinced how a dynamic complex’s bound location can be validated by an average quantity like chemical shift. That might be the reason for apparent difference between predicted and experimental chemical shift prediction. The authors should discuss the reason for the discrepancy in a more clear way.*

**Our response:** Indeed, the comparison and validation is a qualitative one and we have attempted to clarify it in the revised manuscript. In fact, such dynamic complexes are similar to intrinsically disordered domains (like in the *apo*-receptor) that are difficult to characterize with a single structure or even an ensemble of structures resolved by crystallographic and cryo-EM approaches. However, the overall trend is captured by comparisons to the chemical shift differences, giving us increased confidence that indeed the complexes sampled in the simulations are also sampled in the experiments, and more importantly validates the conformational restriction observed in the complex. We would like to reiterate that the work does not directly predict a single structure of the bound complex, but samples the initial steps of chemokine binding and the inherent dynamics associated with complex formation.

4.	*In the related context, the author mention that the ‘to compare this data with simulations reported here, we chose representative structures from each of the coarsegrained simulation and mapped them to their atomistic representation’. The authors should clarify how the representative structure was chosen and how they mapped it to allatom data. In the method section, the authors have cited reference 64 but that is a paper on insane tool. Is this what they meant? Or is it the back-mapping paper by Marrink and coworkers they used? Nonetheless, more details should be provided.*

**Our response:** We backmapped a total of 60 conformations (20 *apo-* and 40 ligand-bound complexes). These were chosen based on the clustering of the populations to sample the conformational space to the greatest extent possible. The receptor conformations were clustered based on the N-terminal backbone conformation. Backmapping was performed using the “Backward” tool and we apologize for this oversight in the reference. We have added the appropriate reference (ref. 78) to the References section in the revised manuscript. The mapped structures were further relaxed through energy minimization and short molecular dynamics simulation runs. We have also updated the Methods section of the revised manuscript, as follows: “*To calculate chemical shift changes in the CXCR1 N-terminal region upon IL8 binding, we considered the main structures sampled and a single conformer from each set was chosen from the highest population cluster. These conformers were transformed to the atomistic description (CHARMM36 force field) using Martini analysis tools^78^. The mapped structures were further equilibrated through energy minimization and short molecular dynamics simulation runs.*” (lines 542-547, p.21 in the revised manuscript).

5.	*The authors should provide a snapshot highlighting ECL1, ECL2, ECL3, Lumen etc in the protein. Otherwise, it is not clear which locations are they referring to. On a related point, the authors should provide a 3D spatial density maps of the encounter of IL8 around protein.*

**Our response:** We thank the reviewer for this suggestion and have accordingly added a schematic (Fig R9, see below). We have highlighted the structural domains resolved experimentally and those that have been modeled in this work. Since the ligand (IL8) is quite large, increased sampling is needed to identify specific pathways. A similar effect is seen in the case of even peptides binding to a related receptor, CXCR4 (reviewed in Kharche and Sengupta, 2020). Based on the reviewer's comments, we plan to build models to identify these pathways as a follow-up of this work. We have included this snapshot as S1 Fig in the revised Supporting Information.

**Fig R9.** Snapshots of three-dimensional structures of CXCR1 and IL8. NMR structure of (a) CXCR1 (PDB ID: 2LNL) with unresolved region of the N-terminus highlighted as a gray tube and (b) interleukin-8 (PDB ID: 1IL8). Extracellular domains of CXCR1 *viz*. ECL1, ECL2, ECL3 and N-terminus are colored in green, light blue, pink and red, respectively. IL8 is shown in cyan, with each region labeled.

6.	*Page 12: The choice of collective variable. The referee understands the reason behind choice of first one i.e backbone RMSD of N-terminal domain. However, it is not Clear how they have chosen the second one. There should be more justification, even if it is merely via visual explanation.*

**Our response:** The second collective variable Met1-Asp26 is used as a reporter of the end-to-end distance and overall shape. The interactions of Met1 would reduce beyond Asp26, and the dynamics of that stretch is constrained due to spatial proximity to secondary structure of helix I. In fact, we projected the population space on the Rg of the N-terminal region (residues 1-26), instead of the distance of Met1-Asp26 (Fig R5, see above) and observe the same trend in both. The parameter Met1-Asp26, therefore, reports not just on that particular residue pair but on the larger conformational changes associated with opening/closing of the N-terminal domain.

7.	*The work report an impressive number of simulation trajectories. This sets an ideal ground for developing a Markov-state-model based analysis for rigorously identifying nd estimating stationary populations of key macro states, predicting eventual binding constants, (on and off) rate constants and the pathways. There are precedences of this. Recently, protein-ligand binding using coarse-grained model has been looked at within the framework of Markov state model. The reviewer understand that this is a formidable job and might itself be an independent work that the authors can seriously think about as a future work to make use of the current trajectories and at least should provide a discussion in the current manuscript.*

**Our response:** We thank the reviewer for the suggestion. As mentioned by the reviewer, the Markov state models constructed to analyze pathways of biomolecular recognition (using the coarse-grain model) have focused on small molecular interactions. We hope to be able to compile the phase space sampled by the receptor here into relative pathways as a next step. We have now extended the Discussion section of the revised manuscript to clarify this point, as follows: “*For a more detailed understanding of the conformational space available in the presence and absence of protein partners, a Markov state model based analysis would be needed for rigorously identifying and estimating stationary populations of key macrostates, binding constants, (on and off) rate constants and the pathways of association. Although this has been previously reported for soluble protein-ligand complexes^63^, it remains challenging for protein-protein complexes involving intrinsically disordered domains.*” (lines 449-455, p.17-18 in the revised manuscript).

**Reviewer #3**

1.	*Kharche et al. has presented a comprehensive simulation-based approach to map the conformational and binding landscape of CXCR1. Even though the approach includes extensive simulations, it lacks the following critical points, which have to be addressed for a publication in Plos Comp Bio.*

**Our response:** We thank the reviewer for the detailed comments and inputs and have taken these into consideration in revising the manuscript.

2.	*The abstract is written in an unclear fashion. The following should be addressed to make it direct and concise.*

**Our response:** We have revised the Abstract to clarify the aspects raised by the reviewer.

3.	*Abstract - Line #35: The sentence starting with "Although .." is too long.*

**Our response:** The sentence has now been modified for brevity (lines 34-36, p.2 in the revised manuscript).

4.	*Abstract - Line #39: What do you mean by "validated atomistic models"? This is unclear, please clarify. Also, you cannot validate your results with NMR Chem Shift predictions, which you derived from your simulations.*

**Our response:** The intrinsic disorder sampled in the *apo* simulations of the coarse-grain model is compared to atomistic models. The conformational restriction of the N-terminal domain upon ligand binding is compared to the NMR chemical shift differences. Since the validation is not quantitative, we have replaced it with the words “*complemented*” and “*corroborated*”, respectively (lines 38 and 41, p.2 in the revised manuscript).

5.	*Abstract - Line #41: The validation by NMR does not include a 100% agreement with the exp and comp data. Please explicitly indicate this in the abstract.*

**Our response:** We have added the phrase "*show that the trends are similar.*" (line 42, p.2 in the revised manuscript) to indicate the lack of 100% quantitative agreement.

6.	*Abstract - Line #43: The authors did not present any data to claim that their simulation data is more reliable than the NMR data. So, how could they claim that NMR data should be used with caution.*

**Our response:** We are sorry for any unintentional miscommunication that could have given rise to this point. We would like to reiterate that NMR chemical shift differences are indeed considered the gold standard to which we compared our predicted values. We observed a good (qualitative) match and overall, the residues with highest chemical shift differences are similar in the simulations and experiments. What we would like to point out here is that residues with the highest chemical shift differences are often considered to the residues at the protein-protein or protein-ligand interface. This correlation between chemical shift difference and contact residues is perhaps true for structured proteins, but may not be a direct match in the case of intrinsically disordered regions (as indicated by our results). The caution we recommend is in the context of interpretation of chemical shift differences, and not the values *per se*, and the direct interpolation of these differences to the identification of binding site residues.

7.	*Introduction - Line #72: The sentence starting with "The high flexibility .." is not clear, please rephrase.*

**Our response:** The sentence has been modified to ensure enhanced clarity (lines 71-73, p.4 in the revised manuscript).

8.	*Introduction - The binding models, the domain organizations & available structures of CXCR1 and IL8 should be illustrated with a figure.*

**Our response:** The starting structures of the simulations that are based on the NMR structure of CXCR1 and the crystal structure of CXCL8 is shown in Fig R9 (see above) together with the domain organization of the receptor. The figure has also been included as S1 Fig in the revised Supporting Information file.

We have extracted representative snapshots for the different binding modes (Fig R10, see below) and added this to the revised Supporting Information as S8 Fig. The corresponding coordinates have been uploaded to the journal site.

**Fig R10.** A schematic representation of the five dominant binding modes of CXCR1-IL8 interactions. The receptor is shown in pink, the N-terminal region is shown in blue, and the IL8 is represented in green.

9.	*Results - Line #414: What are the numbers to come up with the conclusion that "N-terminal relaxed quickly ...". Please quantify.*

**Our response:** The N-terminal domain relaxation is in terms of the extended conformation of the region in the aqueous phase. In the initial placement, the distance of the N-terminal region is far from the membrane (yellow stretches in the plot, see Fig 1d in the revised manuscript), and relaxes in a nanosecond timescale to interact with the membrane (blue stretches). Additionally, we observe the first intra-protein contacts appear immediately (within a nanosecond) and the very extended rigid structure becomes flexible. Keeping in mind the reviewer’s comment, we modified the relevant portions of the Results section, as follows: “*During the simulations, the N-terminal region diverged from the initial structure and appeared to be quite dynamic.*” (lines 142-143, p.6 in the revised manuscript) and “*In the initial placement, the distance of the N-terminal region is far from the membrane (yellow stretches in the plot), and relaxes in a nanosecond timescale to interact with the membrane (blue stretches).*” (lines 147-149, p.6-7 in the revised manuscript).

10.	*Results - What is the population percentages of the two main conformers observed (membrane-bound and receptor-contacted)? How close are the conformations within a single group? Please quantify.*

**Our response:** The values of the receptor “top” conformer are 30%. Please note that these are only orientational states and not conformational states.

11.	*Results - Rg is not very sensitive to group different conformations. Therefore, the authors should present other metrics to convince the reader about the dominant populations observed.*

**Our response:** The two main orientational states of the N-terminal domain are the extended state that interacts with the membrane and the compact state positioned on top of the receptor. Since there are no specific interactions with the receptor ECLs/lumen (due to the dynamics of the N-terminal region), we have used a more general measure such as Rg (and not N-terminal - lumen or ECL contacts). In fact, these orientational states are themselves comprised of multiple conformers, as is evidenced by Fig 4 and S3 Fig. To clarify this, we have explicitly termed these as “*orientational states*” (line 153, p.7 in the revised manuscript) and specified that the “*orientation and position*” of the N-terminal region was highly dynamic (line 154, p.7 in the revised manuscript).

12.	*Results - Line #178: What is the length of the all atom simulation? Did you perform only one simulation? Note this also in the results.*

**Our response:** The contact map shown in the original manuscript was for a single set of atomistic simulation with a 1 μs time course. However, we had previously performed additional simulations (1 μs) of just the N-terminal region with membranes (similar to Kharche *et al.* 2018) that shows a similar conformational sampling. We have now performed additional shorter runs (6*100 ns) starting with different receptor conformers. The updated contact maps are shown in Fig R11 (both for the new simulations and corresponding to all the sets together, see below). We have revised S2 Fig in the Supporting Information to reflect the increased sampling in the all-atom simulations and extended the Methods section accordingly, as follows: “*Atomistic simulations (1 long simulation of 1 μs and 6 sets of 100 ns each from different conformers) totaling to 1.6 μs were carried out as a control.*” (lines 516-517, p.20 in the revised manuscript).

**Fig R11.** Conformational dynamics of the N-terminal region of CXCR1 from all-atom and coarse-grain simulations. Residue-wise contact probabilities of the N-terminal region are plotted for *apo*-CXCR1 in coarse-grain simulations (upper diagonal) and atomistic simulations (lower diagonal), averaged over all simulation sets. The color bar displays probability of interactions between each residue-pair. Panel (a) corresponds to additional atomistic simulations of *apo*-CXCR1, while panel (b) corresponds to a combined sampling from (a) and previous atomistic simulations.

13.	*Results - The authors did not provide any quantitative mean to compare the conformations sampled in the atomistic and coarse-grain simulations. A visual contact map comparison would not be enough here.*

**Our response:** We have refrained from a quantitative comparison as the Martini force-field provides only a semi-quantiative description of the system (discussed in Marrink *et al.* 2004, 2007). In a way, these approaches act as a test of the model itself — for example, if the atomistic simulations sampled stable folded conformers, these would need to be reflected in the coarse-grain simulations as additional distance restrictions. Overall, the intra-protein contacts sampled in the two representations are similar, although a direct comparison is difficult as the distance of closest approach in the coarse-grain model is 0.47 for a regular bead and is much closer in an atomistic model. In general, we observe an RMSD of 0.5-0.6 nm if we perform coarse-grain simulations from a given atomistic structure, due to the underlying distance differences and bead positions. The contact maps are, therefore, provided mainly to corroborate that the conformational dynamics sampled in the coarse-grain and atomistic force-fields are similar.

14.	*Results - I am very skeptical about the approach the authors used to predict CXCR1-IL8 interactions. I am really curious why the authors did not follow the following and tried to predict CXCR1-IL8 interactions with classical MD (which does not seem to fit to their purpose):*

*Isolate the most dominant apo conformers from MD. Gather the available mutagenesis and NMR chem shift data. Use the apo CXCR1-IL8 ensemble, together with the experimental data to predict the binding of CXCR1-IL8 with information-driven docking. Among the available methods, HADDOCK, for example, is perfectly capable of doing this. The authors can then simulate the most viable docking models with MD to analyze their interfaces.*

**Our response:** The main assumption in such a docking-based procedure is that ligand-binding follows a “conformational selection” model and not an “induced fit” model. *A priori*, especially for the N-terminal domain that is highly flexible and unstructured/unresolved in all GPCR structures, one cannot rule out an induced fit model. In addition, protein-protein docking protocols have not been extensively tested with intrinsically disordered domains and both approaches need to be tested.

15.	*Results - Minimum distance between two monomers, as presented in Fig 2a, cannot be a reporter of specific interaction. Please analize the established interfaces in more detail to deduce solid data on binding.*

**Our response:** The binding between CXCR1 and IL8 has been further characterized later in the manuscript. The different binding modes sampled have been shown in Fig 6 of the manuscript and the main interacting residues are shown in Fig 5 of the manuscript. Representative snapshots of the major binding modes are shown in Fig R10 (see above) and have now been included as S8 Fig in the revised Supporting Information.

16.	*Results - Figure 3a: Again here, we need a quantitative comparison.*

**Our response:** The conformational dynamics of the *apo*- and ligand-bound receptors are further characterized in Fig 3 and 4 of the manuscript. The individual contacts in the *apo*- and ligand-bound states are shown in S5 Fig of the revised Supporting Information. As is evident from the data, the conformational state sampled in the *apo*- and the ligand-bound conformers vary.

17.	*Results - Figure 4a: The authors tried to cluster the conformers based on one single distance. They should use different measures (inter-monomer contacts, for example) to see whether their clustering holds. Clustering based on one distance could be misleading.*

**Our response:** We would like to clarify that there is no prior clustering in Fig 4a. Instead, the figure reflects the entire conformational space sampled by the receptor projected on two variables - the inter-residue distance and RMSD (of a reference structure). The distance Met1-Asp26 was used as a reporter of the compactness of the N-terminal domain and the end-to-end distance (Fig R5, see above). The clustering was only performed to identify a reference structure with the highest population, relative to which the entire phase space sampled by the receptor was plotted. We have updated the Methods section to clarify this point, as follows: “*A reference structure was identified from clustering the conformations by pooling all simulation sets in order to project the entire phase space sampled with respect to the RMSD to this reference structure. The clustering was performed using the GROMACS utility gmx cluster using the original GROMOS algorithm implemented in it^77^. The RMSD of the N-terminal region was used to cluster the conformers. The reference structure corresponds to the average structure of highest population cluster I. To analyze the conformational landscape, we projected the population densities along two vectors: RMSD to this reference structure and the distance between residues 1-26. The populations projected on the 2D surface were binned (0.1 nm) and averaged.*” (lines 533-541, p.20-21 in the revised manuscript).

18.	*Results - The authors should present the available experimental data. I could not find their explicit description. For example the agreement between computed and measured NMR data should be number-wise presented in Fig5. Without showing this, it would be bold to claim that simulation data supersedes experimental data and therefore caution should be taken while using experimental data.*

**Our response:** We concur that the chemical shift differences measured in NMR remain the gold standard and would like to reiterate that one of the major take-homes from our present work is that the trend between the predicted and experimental values matches well. Nevertheless, we have added the values of the chemical shift differences (shown in Fig R6, see above) to S7 Fig in the revised Supporting Information. In addition, we would like to point that our suggestion has nothing to do with the experimental data, rather we state that the experimental data should be interpreted with caution. The chemical shift difference reports on the change in the receptor environment upon ligand binding. Consequently, it has usually been directly associated with residues that directly interact with the ligand, and indeed chemical shift changes appear to correlate well with residue-level information for structural domains. However, for intrinsically unstructured domains, such as the N-terminal domain studied here, the conformational restriction upon ligand binding implies that the chemical shift differences report on the conformational space as well. We, therefore, recommend that the extrapolation of chemical shift difference data to interacting residues should be done with caution, and do not comment at all on the actual chemical shift values. To clarify this point further, we have now reworded the relevant portions of the Abstract and Discussion sections in the revised manuscript, as follows: “*Interestingly, our results indicate that the residues with maximum interactions do not necessarily exhibit the highest chemical shift perturbation. In the case of intrinsically disordered regions, there may not be a direct correlation between residues with high chemical shift perturbations and those at the interface of the receptor-ligand contacts.^51^*” (lines 427-430, p.17 in the revised manuscript).

19.	*Discussion - Should be updated according to the new findings.*

**Our response:** Our work provides an overall understanding of conformational dynamics of the N-terminal region, and the reduced conformational space upon ligand binding. We have revised the relevant parts of the Discussion section to clarify the points raised by the reviewer.

20.	*Methods - What is the force field used during atomistic simulation? Also, how many simulations were performed here? If the authors performed one simulation, then they should run more.*

**Our response:** The atomistic simulations were performed using the CHARMM36 force-field. We have updated the Methods section to clarify this oversight (line 511, p.20 in the revised manuscript). The contact map shown in the original manuscript was a single set of atomistic simulation with a 1 μs time course. However, we have now performed additional simulations (1 μs) of just the N-terminal domain with membranes (similar to Kharche *et al.* 2018) that shows a similar conformational sampling. We have performed additional shorter runs (600 ns) with different starting receptor conformations (Fig R11, see above). We have revised S2 Fig in the revised Supporting Information to reflect the increased sampling in the all atom simulations and extended the Methods section, as follows: “*Atomistic simulations (1 long simulation of 1 μs and 6 sets of 100 ns each from different conformers) totaling to 1.6 μs were carried out as a control.*” (lines 516-517, p.20 in the revised manuscript).

21.	*Methods - Why did the authors use a such an old version of GROMACS?*

**Our response:** The simulations were performed to be consistent with our previous work on rabbit CXCR1 N-terminal construct and subsequent analysis of the N-terminal domain dynamics. We have now tested a newer version of GROMACS 5.1.4 and GROMACS 2020.

**Reviewer #4**

1.	*This manuscript uses extensive CG (and some atomistic) simulations to study the interactions of the key chemokine receptor CXCR1 with its ligand IL8, which serves to regulate innate immune responses.*

*The N-terminal region of the receptor is thought to bind to the ligand (similarly to other GPCRs), but this is currently structurally unresolved. Thus, CG simulations were used to predict possible N-terminal conformations in the context of a membrane environment and ligand bound states.*

*The key results are that IL8 stabilises the otherwise highly flexible / disordered N-terminus. Results were validated to a certain extent by comparison with atomistic simulations and prior NMR chemical shift and mutagenesis studies.*

*Overall, the work will be of interest to the membrane receptor community, and might also be useful for future drug design efforts (though this isn’t discussed).*

**Our response:** We thank the reviewer for the positive comments which we appreciate. Indeed, we believe that our results open up a larger perspective for therapeutic design for drugs targeting intrinsically disordered domains. We are currently testing if these design principles can be generalized to the receptor subfamily. Keeping this in mind, we have extended the Discussion section in the revised manuscript to reflect these translational aspects of our current work, as follows: “*An emerging theme from the current work is its general relevance to intrinsically disordered domains, especially in the context of therapeutic design.*” (lines 446-447, p.17 in the revised manuscript).

2.	*The key focus here is the N-terminal region of CXCR1, and all the results depend on how this was treated. The CG simulations were performed starting from an extended N-terminal state, which was mapped to CG using Martini 2.1. It’s good that the authors provide their input files, but for the casual reader, the authors should say more about how the structure was treated in the manuscript. E.g. just angles/dihedrals (if so, did that result in a uniform set of extended secondary structure parameters?), or was an elastic network also implemented?*

**Our response:** The N-terminal domain of the receptor was treated with standard Martini parameters. The elastic network was implemented for the chemokine CXCL8 core and in one set of simulations for the transmembrane domain. Indeed, the N-terminal domain relaxes into the compacted dynamic conformational ensemble in the absence of any added secondary structure parameters. In fact, our first test simulations were with atomistic models of the N-terminal construct (in the presence of a membrane) and the full-length receptor. Since we were unable to sample a stable secondary structure, we were able to proceed with the coarse-grain force-field with only the standard parameters (no elastic network on the N-terminal region). The parameters used are included in the files deposited to the journal site.

3.	*The authors conclude that the N-terminus samples similar conformations in CG and atomistic simulations (Fig 1 vs Fig S1). However, I would like to see e.g. the minimum distances and radii of gyration for the atomistic resolution. If things are not reproduced across resolutions perfectly, that’s okay, but it would be useful to know for other researchers in the field. It would also be useful for other researchers to know if they could just use the Martini forcefield “out of the box” in future to model such N-terminal regions in GPCRs, or if more extensive refinement against atomistic data might first be required.*

**Our response:** We believe that the Martini force-field should not be used “out of the box” and that each system needs to be tested in a case-by-case basis. For domains that exhibit folded conformations, the Martini secondary structural constraints (elastic network) would need to be added. Perhaps the Go-model implemented in the newer version of the force-field would be able to handle the conformational dynamics better. In this particular case, it would be difficult to directly match the minimum distance, as these are set to 0.47 nm for the standard bead size in the coarse-grain force-field and are atom-dependent in the atomistic models. Similarly, the Rg values are not directly comparable. As a result, we have instead generated contact maps that report on the overall conformational states available. Please note that the cutoffs for the coarse-grain and atomistic models vary, and the values normalized to the simulation length/sets could be directly compared. These are given on the two sides of the matrix (upper diagonal: coarse-grain and lower diagonal: atomistic) in Fig 3 in the revised manuscript. A more detailed comparison is difficult at this stage.

4.	*How much faith do the authors have in the CG simulations in correctly predicting conformational changes in the N-terminal domain upon ligand binding? CG snapshots were converted to atomistic resolution, and then used to compute chemical shifts for comparison with experiments. But it might be expected that the method of conversion to atomistic representation may well affect this calculation; what would happen if the CG snapshots were first refined (even for just a few nanoseconds) in atomistic simulations? The authors warn that caution should be exercised when interpreting chemical shift perturbation data, which I agree with, but not just because of the experimental limitations.*

**Our response:** We have provided a more quantitative comparison to the experimental measures. We agree that the match is not quantitative and only the trends can be compared. We would like to point out that we do not recommend caution about the use of the chemical shift difference data, only the interpretation that it could be directly used to identify the “binding” residues. Our results indicate that the conformational restriction experienced by the N-terminal domain in the ligand-bound state also contributes to the chemical shift differences.

5.	*The authors point out that both receptors and chemokines have been shown to dimerize in vivo – it would be good to discuss how this might affect the observations reported here.*

**Our response:** Indeed, as also pointed out by Reviewer 1, the dimerization of the receptor could alter ligand binding. However, the 1:1 stoichiometry (1 receptor:1 ligand) has been reported to probably be the predominant signaling form. The dimer interface of CXCR1 is not yet known, but the crystal structure of CXCR4 suggests a dimer interface formed by transmembrane helices IV and V. Since transmembrane helix I faces outward, the dynamics of N-terminal domain would not be directly constrained. We have extended the discussion in the revised manuscript accordingly, as follows: “*An important point to consider would be the dimerization of the receptor and the ligand could alter ligand binding. However, the 1:1 stoichiometry (1 receptor:1 ligand) has been reported to probably be the predominant signaling form^24-26^.*” (lines 455-458, p.18 in the revised manuscript).

6.	*Likewise, as noted by the authors, the loops of GPCRs are sensitive to lipid types. Simulations reported here were of simple POPC lipid membranes – do the authors expect that more realistic complex lipid compositions might affect their observations?*

**Our response:** We thank the reviewer for the comment. Indeed, that is the next step and we are currently working on a more complex membrane. In addition, we have touched upon this important aspect in the revised manuscript, as follows: “*Another limitation of the current work is the simple membrane model considered. However, several of the experimental studies (fluorescence, NMR) have been performed in model membranes or even detergent micelles. The main salient features of the interaction are not suggested to differ, although the binding would be modulated overall.*” (lines 458-461, p.18 in the revised manuscript).

7.	*Would the authors be able to comment on whether their derived conformations might be useful in drug design? And for that matter, it would be nice if the authors made some of their dominant ligand bound structures available to the community.*

**Our response:** The N-terminal domain of GPCRs, and chemokine receptors in particular, indeed play a large role in ligand specificity. However, for small molecular drug design, the ligands appear to bind in the lumen of the receptor, independent of the N-terminal (based on available structures of chemokine receptors). However, we agree that the main ligand bound structures should be provided and have uploaded them to the journal site.

**Additional journal instructions**

1.	*Figure Files:* We have checked the files on PACE and uploaded the modified files.

2.	*Data requirements:* We have uploaded the input files and some of the output files, *i.e.*, the binding modes.

3.	*Reproducibility:* Not applicable.

Many thanks for your cooperation during the review process. I hope you would find our revised manuscript satisfactory. I remain eager to hear from you.

Yours sincerely,

**Prof. Amitabha Chattopadhyay**

SERB Distinguished Fellow

CSIR-Centre for Cellular and Molecular Biology

Uppal Road, Hyderabad 500 007

India

Phone: +91-40-2719-2578, Fax: +91-40-2716-0311

E-mail: amit@ccmb.res.in

URL: http://e-portal.ccmb.res.in/e-space/amit/Pages/Index.htm

**References**

Daura X, Gademann K, Jaun B, Seebach D, van Gunsteren WF, Mark AE. Peptide folding: when simulation meets experiment. Angew Chem Int Ed. 1999;38: 236-240.

Kharche SA, Sengupta D. Dynamic protein interfaces and conformational landscapes of membrane protein complexes. Curr Opin Struct Biol. 2020;61: 191-197.

Kharche S, Joshi M, Sengupta D, Chattopadhyay A. Membrane-induced organization and dynamics of the N-terminal domain of chemokine receptor CXCR1: insights from atomistic simulations. Chem Phys Lipids. 2018;210: 142-148.

Marrink SJ, de Vries AH, Mark AE. Coarse grained model for semiquantitative lipid simulations. J Phys Chem B. 2004;108: 750-760.

Marrink SJ, Risselada HJ, Yefimov S, Tieleman DP, de Vries AH. The MARTINI force field: coarse grained model for biomolecular simulations. J Phys Chem B. 2007;111: 7812-7824.
